# Profiling of chromatin accessibility and identification of general *cis*-regulatory mechanisms that control two ocular lens differentiation pathways

**DOI:** 10.1186/s13072-019-0272-y

**Published:** 2019-05-03

**Authors:** Yilin Zhao, Deyou Zheng, Ales Cvekl

**Affiliations:** 1 The Departments of Genetics, Albert Einstein College of Medicine, Bronx, NY 10461 USA; 2 Neurology and Neuroscience, Albert Einstein College of Medicine, Bronx, NY 10461 USA; 3 Ophthalmology and Visual Sciences, Albert Einstein College of Medicine, Bronx, NY 10461 USA

**Keywords:** ATAC-seq, Differentiation, Lens, “Open” chromatin, RNA-seq, Tissue specificity

## Abstract

**Background:**

Promoters and enhancers are *cis*-regulatory DNA sequences that control specificity and quantity of transcription. Both are rich on clusters of *cis*-acting sites that interact with sequence-specific DNA-binding transcription factors (TFs). At the level of chromatin, these regions display increased nuclease sensitivity, reduced nucleosome density, including nucleosome-free regions, and specific combinations of posttranslational modifications of core histone proteins. Together, “open” and “closed” chromatins represent transcriptionally active and repressed states of individual genes, respectively. Cellular differentiation is marked by changes in local chromatin structure. Lens morphogenesis, regulated by TF Pax6, includes differentiation of epithelial precursor cells into lens fibers in parallel with differentiation of epithelial precursors into the mature lens epithelium.

**Results:**

Using ATAC-seq, we investigated dynamics of chromatin changes during mouse lens fibers and epithelium differentiation. Tissue-specific features of these processes are demonstrated via comparative studies of embryonic stem cells, forebrain, and liver chromatins. Unbiased analysis reveals *cis*-regulatory logic of lens differentiation through known (e.g., AP-1, Ets, Hsf4, Maf, and Pax6 sites) and novel (e.g., CTCF, Tead, and NF1) motifs. Twenty-six DNA-binding TFs, recognizing these *cis*-motifs, are markedly up-regulated in differentiating lens fibers. As specific examples, our ATAC-seq data uncovered both the regulatory regions and TF binding motifs in *Foxe3*, *Prox1*, and *Mip* loci that are consistent with previous, though incomplete, experimental data. A cross-examination of Pax6 binding with ATAC-seq data demonstrated that Pax6 bound to both open (H3K27ac and P300-enriched) and closed chromatin domains in lens and forebrain.

**Conclusions:**

Our study has generated the first lens chromatin accessibility maps that support a general model of stage-specific chromatin changes associated with transcriptional activities of batteries of genes required for lens fiber cell formation. Analysis of active (or open) promoters and enhancers reveals important *cis*-DNA motifs that establish the molecular foundation for temporally and spatially regulated gene expression in lens. Together, our data and models open new avenues for the field to conduct mechanistic studies of transcriptional control regions, reconstruction of gene regulatory networks that govern lens morphogenesis, and identification of cataract-causing mutations in noncoding sequences.

**Electronic supplementary material:**

The online version of this article (10.1186/s13072-019-0272-y) contains supplementary material, which is available to authorized users.

## Introduction

Cellular differentiation is driven by the coordinated expression of batteries of genes that encode proteins required for cellular specialization and function. Differentiation is mostly regulated at the level of transcription. Tissue specificity of transcription is primarily regulated by a combinatorial action of sequence-specific DNA-binding transcription factors and their interactions with promoters and distal enhancers [1, 2]. Both promoters and enhancers of transcriptionally active genes display increased sensitivity to nuclease digestion [3] and are located to “open” chromatin domains. Open chromatin regions display lower nucleosomal density or even nucleosome-free regions [2]. In addition, active chromatin is marked by a specific combination of modified core histone proteins (H3K27ac, H3K4me1, and H3K4me3) and by the presence of H2A.Z and H3.3 histone variants [4–6]. In contrast, transcriptionally repressed genes are often organized within “closed” chromatin domains, marked by different histone modifications (e.g., H3K27me3 and H3K9me3) [2, 7]. Hence, distinct cell types as well as differentiation cascades yielding differentiated cells display unique chromatin structure. Studies of chromatin dynamics, such as changes in open chromatin regions during differentiation and between different cell types, thus provide critical insights into the molecular mechanisms of tissue specialization and differentiation.

**Fig. 1:**
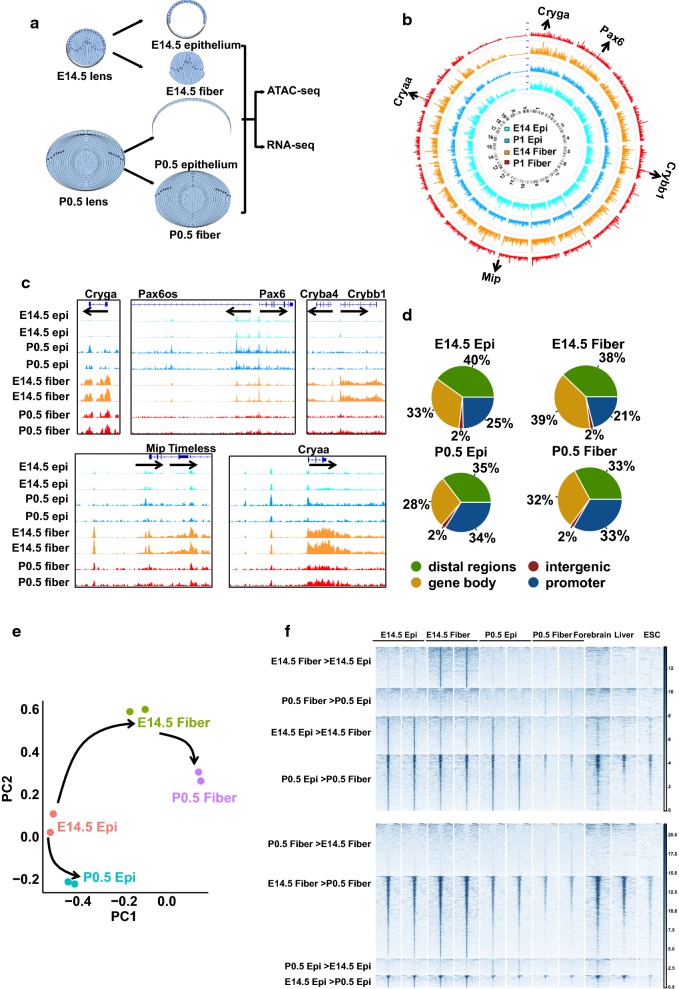
ATAC-seq analysis of lens fibers and lens epithelium. **a** Schematic diagram of embryonic E14.5 and newborn (P0.5) lens morphology. **b** Circos plot of global chromatin accessibility in all ATAC-seq samples (mean read counts inside peaks from biological replicates normalized by all read numbers inside all peaks). The arrows mark some genes with highest peaks in E14.5 fibers. **c** ATAC-seq signal tracks of the genes marked in panel **b**. **d** Pie charts show the genomic distributions of peaks. **e** Principal component analysis of the top 500 peaks with the biggest variance across 8 samples. The arrows show lens fiber cell differentiation (E14.5 epithelium (epi) → E14.5 fibers → P0.5 fibers) and epithelium maturation (E14.5 epi → P0.5 epi) paths. **f** Heat maps show the read densities of lens, forebrain, liver, and ESCs ATAC-seq data within ± 5 kb from the centers of differential accessible regions (DARs) which are from the pairwise comparison. Epi and fiber represent epithelial and fiber cells

The ocular lens is a highly specialized tissue that is formed from a single lens progenitor cell type. The progenitors give rise to the lens vesicle, comprised of lens precursor cells that ultimately differentiate into lens epithelial and lens fiber cells [8, 9]. Thus, lens differentiation is an excellent model to study early (i.e., formation of lens progenitors), middle (i.e., formation of lens fibers and lens epithelium), and late stages of differentiation (maturation of epithelium and terminal differentiation–maturation of lens fibers including degradation of their nuclei) [10]. Mouse lens differentiation is the leading model for understanding the complexity of these processes [11]. The lens precursor cells form a transitional polarized structure, the hollow lens vesicle in E11.5 mouse embryos. Its anterior part differentiates into the lens epithelium while its posterior part differentiates into the “primary” lens fibers to fill up the space by E14.5 of mouse embryogenesis (Fig. 1a). The bulk of the mature lens is formed by highly elongated lens fibers that represent cells at the terminal differentiation state. From E14.5, lens growth is driven by a proliferating subpopulation of the lens epithelium (“germinative zone”) that exits the cell cycle at the lens equator and generates “secondary” lens fibers [8, 9]. The newborn (P0.5) mouse lens is comprised of approximately 30,000 epithelial and over 140,000 lens fiber cells (Fig. 1a) [9, 12]. Recent transcriptome analysis of both lens epithelium and fibers by RNA-seq revealed thousands of differentially expressed genes (DEGs) between these two compartments, including a number of highly lens-specific genes [13]. Analysis of transcription factors (TFs) and their DNA-binding motifs among the differentially expressed genes confirmed pivotal roles of many known TFs and implicated novel TFs that may help drive lens differentiation. A major question in the field is to link the expression data with changes in chromatin structure. In turn, the analysis of chromatin accessibility would allow genome-wide identification of promoter regions and distal enhancers and unbiased analysis of the *cis*-regulatory grammar [2] of the lens-specific gene expression programs.

The entire process of lens differentiation, from the birth of individual lens progenitor cells to the terminal differentiation of lens fibers and massive up-regulation of crystallin gene expression, is directly or indirectly regulated by the transcription factor Pax6 [10, 14, 15]. Pax6 acts as a dual transcriptional activator and repressor [16] through interactions with and recruitment of different chromatin remodeling complexes [17]. Other well-studied transcription factors of lens differentiation include c-Maf, FoxE3, Gata3, Hsf4, Mab21l1, Msx2, Pitx3, Prox1, Sox1, Sox2, Sox11, Tfap2a (AP-2α), and Zeb2 (Sip1) [11]. More recent studies point to the involvement of transcription factors downstream of BMP, FGF, Hippo-Yap, integrin, and Notch signaling [11]. Nevertheless, the regulatory mechanisms and genome-wide targets of these transcription factors remain poorly understood in lens differentiation.

Recent advances in genome technologies greatly simplify the identification of open chromatin domains using a single assay, the ATAC-seq [18]. Using this method, a series of recent studies have made novel insights into the dynamics of open chromatin during early lineage commitments and terminal differentiation [19–22]. In the present study, we aimed to investigate dynamic changes in chromatin structure during lens differentiation and employed recently published datasets of non-lens cells for comparative studies to examine the detected open chromatin regions. We started with the E14.5 lens, which allows precise separation of the nascent lens epithelium and lens fiber mass. We also linked our new ATAC-seq data to our previously published RNA-seq data. Likewise, we also analyzed P0.5 lens, which represents a stage comprised from easily separable mature lens epithelium and lens fibers. We further compared our lens ATAC-seq data with other publicly available data to achieve more comprehensive understandings of lens open chromatin dynamics and their functions in lens differentiation.

## Results

### Identification of open chromatin domains in E14.5 and P0.5 lens epithelium and lens fibers by ATAC-seq

To identify and characterize chromatin dynamics during mouse lens differentiation, we collected E14.5 and P0.5 lens epithelial and fiber cells through micro-dissection (Fig. 1a). The isolated nuclei were used to construct ATAC-seq libraries in duplicates for sequencing, following an established protocol [18]. We obtained an average of 24.5 million read pairs per sample after filtering, and their alignment rates to the mouse genome were over 96% (Additional file 1: Table S1). We next called peaks for each sample using the software MACS2 (v 2.1.0) [23] and got > 100,000 and ~ 60,000 peaks for E14.5 and P0.5 samples, respectively, with adjusted *p* values (*q*) < 0.05 as cutoff (Additional file 1: Table S1). After filtering out peaks in the mouse genome blacklist regions [24], we identified a total number of 185,297 peaks across all eight samples. Importantly, ATAC-seq data showed high correlations between all biological replicates (Pearson correlation coefficients (*r*) > 0.95, Additional file 2: Fig S1), indicating highly reproducible data. We first used the Circos plots to visualize global chromatin accessibility and illuminate differences among all samples (Fig. 1b). This global visualization and analyses of individual peaks revealed a global reduction of chromatin accessibility from E14.5 to P0.5 in both lens epithelium and fibers (Additional file 2: Fig S1). We further examined specific regions with high ATAC-seq signals. Notably, we found that they were mapped to both critical regulatory (e.g., Pax6) and structural (e.g., Cryaa, Crybb1, Cryga, and Mip/aquaporin0; Fig. 1c) genes of the lens morphogenesis. Some of those ATAC-seq peaks were cell type specific while other peaks were spatially and temporally shared. For example, Cryga, Cryba4, Crybb1, Mip, and Cryaa showed peaks with higher signals in lens fibers while Pax6 showed peaks with higher signals in lens epithelium. We next examined the distributions of peaks at promoters, gene bodies, intergenic regions, and distal regions (Fig. 1d). P0.5 epithelium and fibers showed a higher percentage of peaks mapped to promoters than E14.5 epithelium and fibers (*p* = 0.21 for percentages of promoter peaks in E14.5 vs. P0.5 epithelium and *p* = 0.08 for E14.5 vs. P0.5 fibers; Chi-squared test). Principal component analysis of the top 500 peaks with biggest variances separated the eight samples into four groups and organized them into two differentiation pathways that matched the known biology of lens differentiation (Fig. 1e): lens fiber cell differentiation (E14.5 epithelium → E14.5 fibers → P0.5 fibers) and epithelium maturation (E14.5 epithelium → P0.5 epithelium).

To identify the regions with spatially or temporally differential chromatin accessibilities, we conducted four pairwise comparisons (E14.5 epithelium vs. E14.5 fibers, P0.5 epithelium vs. P0.5 fibers, E14.5 epithelium vs. P0.5 epithelium, and E14.5 fibers vs. P0.5 fibers). We detected thousands of differentially accessible regions (DARs) using EdgeR [25] by the following parameters: false discovery rate (FDR) < 0.05, counts per million mapped reads (cpm) > 2, and fold change (FC) > 2 (Additional files 3 and 4: Table S2 and Fig. S2). Finally, we generated comparative heat maps of ATAC-seq read densities at ± 5 kb from DARs centers to visualize the patterns of chromatin in lens. To find out whether those DARs are open in non-lens cell types, we also included ATCA-seq data of E14.5 forebrain (ENCSR810HQR), E14.5 liver (ENCSR032HKE), and ESCs (GSE66390) [26] downloaded from ENCODE [24, 27] and GEO [28]. Most notably, the peaks with higher signals in fibers than in epithelium at E14.5 and P0.5 did not show any signals in forebrain, liver, and ESCs chromatins. On the contrary, some of the peaks with higher signals in epithelium than in fibers at both E14.5 and P0.5 also showed ATAC-seq signal enrichments in forebrain, liver, and ESCs samples. Temporally, the peaks with higher signals in P0.5 were absent in forebrain, liver, and ESCs. However, the peaks with higher signals in E14.5 showed clear signals in other cell types (Fig. 1f). Taken together, these analyses both demonstrate quality and reproducibility of our lens ATAC-seq data and reveal numerous lens open chromatin regions that are closed in forebrain, liver, and ESCs.

### Characterization of eight chromatin accessibility patterns through lens development

**Fig. 2:**
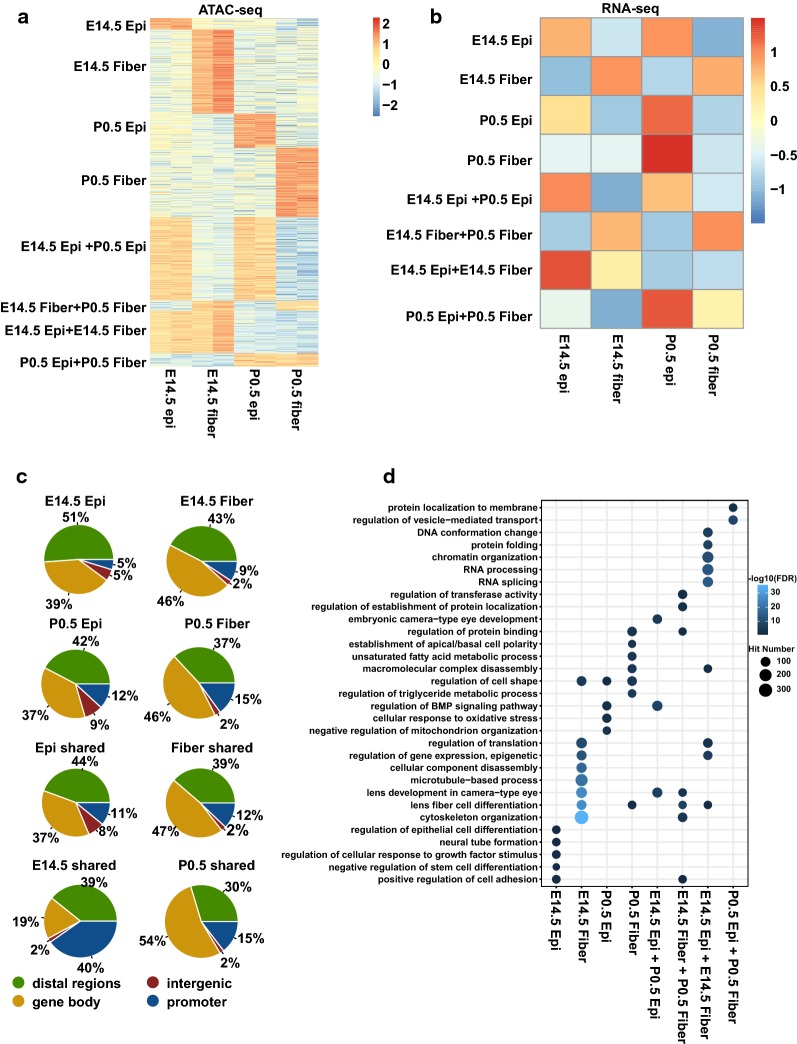
Analysis of unique or shared clusters of open chromatin regions. **a** Heat map shows ATAC-seq signals for unique and shared peak groups. **b** Heat map shows median expression of the genes associated with the top 100 most different peaks (ranked by fold change). **c** Pie charts show the genomic distributions of the unique and shared peaks. **d** Dot plot shows the enriched GO terms for each group of peaks. The dot color represents –log10(FDR), and the dot size represents number of regions in the GO terms

To better understand lens chromatin dynamics, we first identified the open chromatin regions unique to each sample and then defined regions either shared temporally between E14.5 and P0.5 at epithelium or fibers or spatially between epithelium and fibers (Fig. 2a, Additional file 5: Table S3). We further evaluated the median RNA expression levels of genes associated with the top 100 peaks (ranked by fold changes) using our previous RNA-seq data [29]. This analysis found that the expression patterns of the genes with shared ATAC-seq peaks were similar to the patterns of their ATAC-seq signal differences. However, the genes with unique peaks did not exhibit expression patterns mirroring the uniqueness in chromatin accessibilities (Fig. 2b, Additional file 6: Fig. S3). We thus examined the genomic distributions of the unique and shared peaks and found a few notable differences. For example, compared to the groups of peaks with higher signals in E14.5 epithelium or fibers, the peaks with higher signals in P0.5 epithelium and fibers had higher percentages of promoter peaks (*p* = 0.13 for percentages of promoter peaks in E14.5 vs. P0.5 epithelium, *p* = 0.28 for E14.5 vs. P0.5 fibers, Chi-squared test) (Fig. 2c). This finding prompted us to perform an unbiased functional classification of gene groups with distinct patterns of ATAC-seq peaks.

We thus analyzed the enrichments of gene ontologies (GO) for each cluster of peaks using the GREAT software (v 3.0.0) [30]. We found that individual clusters of peaks were enriched for genes of various functions but all related to cellular differentiation and tissue remodeling. For example, “regulation of epithelial cell differentiation,” and “negative regulation of stem cell differentiation” were enriched in the peaks that were unique in E14.5 epithelium, while “cytoskeleton organization” and “lens fiber cell differentiation” were enriched in the peaks unique in E14.5 fibers. “Regulation of BMP signaling pathway” term was enriched in the peak group with higher signals in P0.5 epithelium as well as the peaks shared between the E14.5 and P0.5 epithelium (Fig. 2d). Importantly, these findings are fully consistent with current knowledge of lens differentiation.

**Fig. 3:**
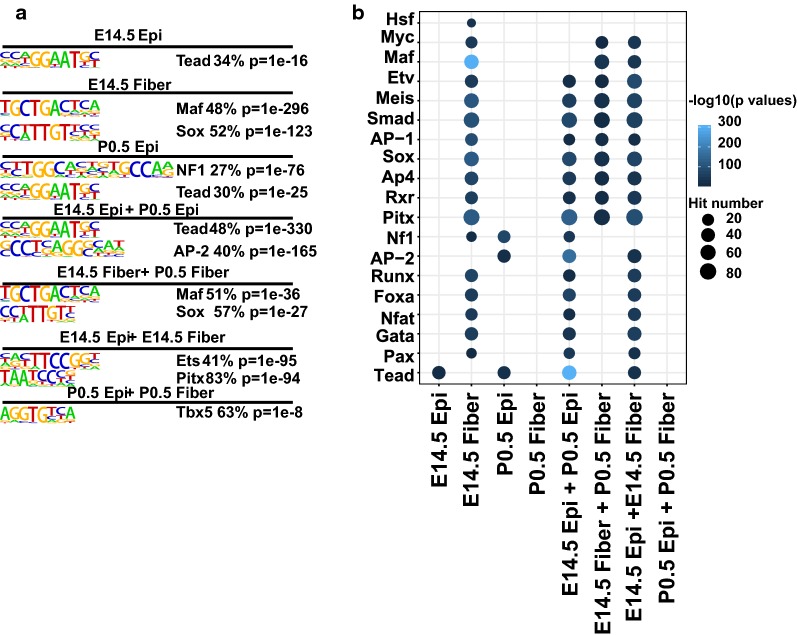
Motif analysis in unique and shared clusters of open chromatin regions. **a** Top enriched motifs in each group of unique and shared peaks. **b** Dot plot shows enrichment of motifs in each group of peaks. The dot color represents –log10(*p* value), and the dot size represents the percentages of peaks with the motifs

To probe whether the patterns of co-regulated gene expression could be directly linked to the *cis*-regulatory grammar of transcription, we next searched for the transcription factor binding motifs that were significantly enriched inside each cluster in relation to all ATAC-seq peaks using the HOMER (v 4.7) software [31]. The Tead motif was the top enriched *cis*-element in the group of peaks with highest signals in E14.5 epithelium as well as within the peaks shared between E14.5 and P0.5 epithelium. The Maf and Sox motifs were the top motifs enriched in the peaks unique in E14.5 fibers and the shared peaks in E14.5 and P0.5 fibers (Fig. 3a). We also found the Pax, Gata, Nfat, Fox, Runx motifs enriched in the peaks unique in E14.5 fibers, the shared peaks in E14.5 epithelium and P0.5 epithelium, as well as in the shared peaks in E14.5 epithelium and E14.5 fibers, indicating their functional involvements at lens epithelium and early stages of lens development. In contrast, the Pitx, RXR, Ap4, Sox, AP-1, Smad, Meis, and Etv motifs were enriched in multiple peak groups, including the peaks shared by E14.5 and P0.5 epithelium and the peaks shared by E14.5 and P0.5 fibers, indicating their roles in both lens compartments (Fig. 3b). Taken together, these analyses shed new light on the underlying regulatory mechanisms and the employment of both established and novel transcription factors during lens development.

### Chromatin accessibility versus gene expression changes: in-depth analysis

To further explore our data, we next mapped all the DARs to their adjacent genes. Unexpectedly, we found that only 13–29% of DARs were mapped to previously detected differentially expressed genes (DEGs) [29] (Additional file 4: Table S2 and Additional file 7: Fig. S4a). The majority of DARs (55–78%) were assigned to genes that were not differentially expressed, while 9–29% of DARs were assigned to the genes that showed expression patterns opposite to chromatin changes (Additional file 7: Fig. S4a). Nevertheless, a close examination of individual groups of DARs showed that the percentages of DARs assigned to DEGs were associated with the numbers of DARs annotated to genes. More specifically, genes with a higher number of DARs were more likely to be differentially expressed (Additional file 7: Fig. S4b). Therefore, we conclude that the DARs identified here are overall correlated with gene expression changes.

**Fig. 4:**
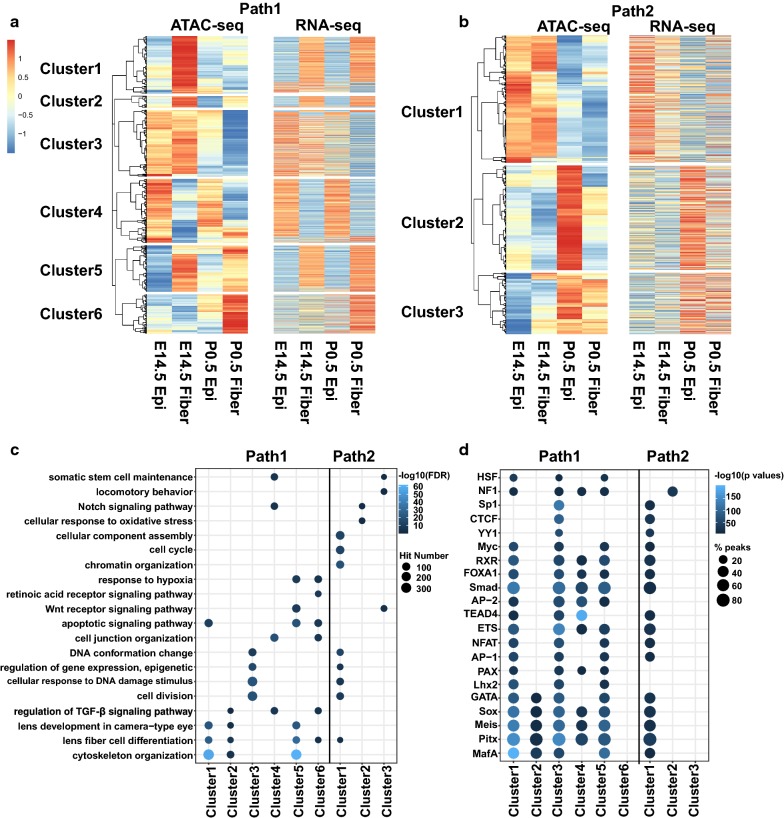
Functional analysis of DARs at DEGs. **a**, **b** Heat maps show ATAC-seq read densities and gene expression by RNA-seq for DEGs in the 6 clusters of peaks in Path1 (lens fiber cell differentiation path) (**a**) and 3 clusters of Path2 (lens epithelium maturation path) (**b**). **c**, **d** Dot plots show enriched GO terms and TF motifs for the DAR clusters of Path1 and Path2. The dot color represents –log10(FDR) or –log10(*p* value), and the dot size represents number of regions enriched in the GO terms or percentages of peaks with the motifs

To further examine DARs in the context of lens differentiation, we divided all the DARs at DEGs into “lens fiber cell differentiation path” (Path1, E14.5 epithelium → E14.5 fibers → P0.5 fibers) and “lens epithelium maturation path” (Path2, E14.5 epithelium → P0.5 epithelium). In total, we collected 14,768 ATAC-seq peaks (mapped to 5249 DEGs detected in RNA-seq) and 1810 peaks (mapped to 1318 DEGs detected in RNA-seq) in the Path1 and Path2, respectively. Using hierarchical clustering of the ATAC-seq signals, we could divide the peaks in the lens fibers and epithelium differentiation paths into 6 and 3 clusters, respectively (Fig. 4a, b). For example, the cluster 1 in Path1 (2980 peaks) represented the chromatin regions closed at E14.5 epithelium, open at E14.5 fibers, and then closed at P0.5 fibers. Likewise, the “bottom” cluster 6 represented mostly closed regions at E14.5 epithelium, E14.5 fibers, and P0.5 epithelium and open regions in P0.5 fibers. Notably, the patterns of open and closed ATAC-seq signals for each cluster matched with the high and low expression of the corresponding genes (Fig. 4a, b).

We thus re-examined the enriched GO terms and TF motifs for individual clusters of ATAC-seq peaks that were mapped to DEGs, as described above for the global analysis. We found distinct GO terms and motifs; however, this time the results probed deeper layers of the lens differentiation processes. For example, the Path1 cluster 1 peaks were enriched for GO terms of “lens fiber cell differentiation” and “cytoskeleton organization.” The Path1 cluster 3 and the Path2 cluster 1 were both enriched for “DNA conformation change,” “regulation of gene expression, epigenetic,” and “cell division.” The Path1 clusters 2, 4, and 6 were enriched for “regulation of TGFβ signaling pathway” (Fig. 4c). For the TF motif analysis, the majority of *cis*-sites detected were shared between the Path1 and Path2, demonstrating both the unity and hierarchical structure of gene programs for lens differentiation processes (Fig. 4d). The top TF motifs predicted above (Maf, Sox, Tead, NF1, Ets, and Pitx, see Fig. 2d) were detected again using DARs at DEGs. Interestingly, the Pax and Lhx motifs were found enriched at the Path1 clusters 1, 3, and 5. In contrast, both the Path1 cluster 6 and the Path2 cluster 3 yielded almost no common motifs, respectively. These findings indicate that some lens differentiation pathways are coordinately regulated by common *cis*-motifs and TF factors while others potentially employ more diverse repertoires of *cis*-sites that cannot be readily detected computationally.

**Fig. 5:**
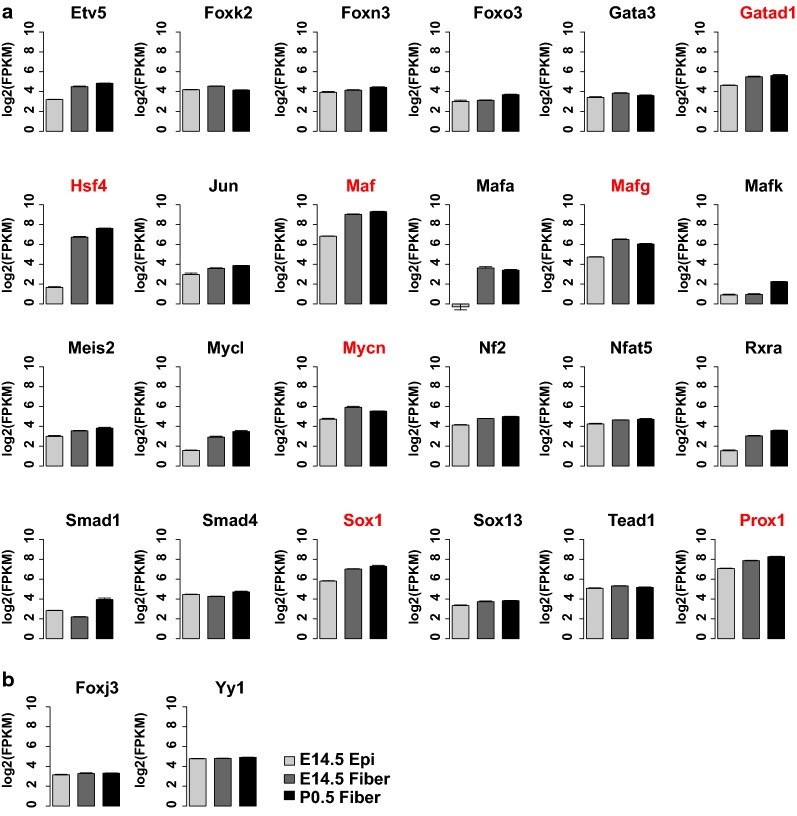
Expression profiles of selected transcription factors linked to lens fiber cell differentiation (Path1). **a**, **b** Bar plots show the log2(FPKM) of the transcription factors at E14.5 epithelium, E14.5 fibers, and P0.5 fibers. The red color indicates highly expressed TFs (FPKM > 50). **a** TFs with significantly increased expression in fiber cell differentiation path (in E14.5 epithelium vs. E14.5 fibers or E14.5 fibers vs. P0.5 fibers comparisons). **b** TFs not significantly differentially expressed during fiber cell differentiation path

To identify functionally relevant TFs, we examined steady-state expression levels of individual mRNAs encoding TFs known to recognize the *cis*-motifs summarized in Fig. 4d. We first used the hierarchical clustering method to divide the TFs into Path1 and Path2 based on their expression (Additional file 8: Fig. S5a and b). In Path1, we provide a summary of 27 transcription factors, 25 of which were significantly up-regulated in the fiber cell differentiation pathway (FDR < 0.05 in E14.5 epithelium vs. E14.5 fibers or E14.5 fibers vs. P0.5 fibers) (Fig. 5; Sox8 and Nfatc1 not shown due to low expression). Although motifs for the highly abundant transcription factor Prox1 were not found (see Discussion), we identified ATAC-seq peaks at previously described Prox1 binding regions at the promoters of multiple genes important for lens function, including *Fgfr3*, *Fgfrl1*, *Lctl*, and *Crybb1* (Additional file 9: Fig. S6) [32, 33]. Thus, Prox1 expression profile was added here for its importance (Fig. 5a).

**Fig. 6:**
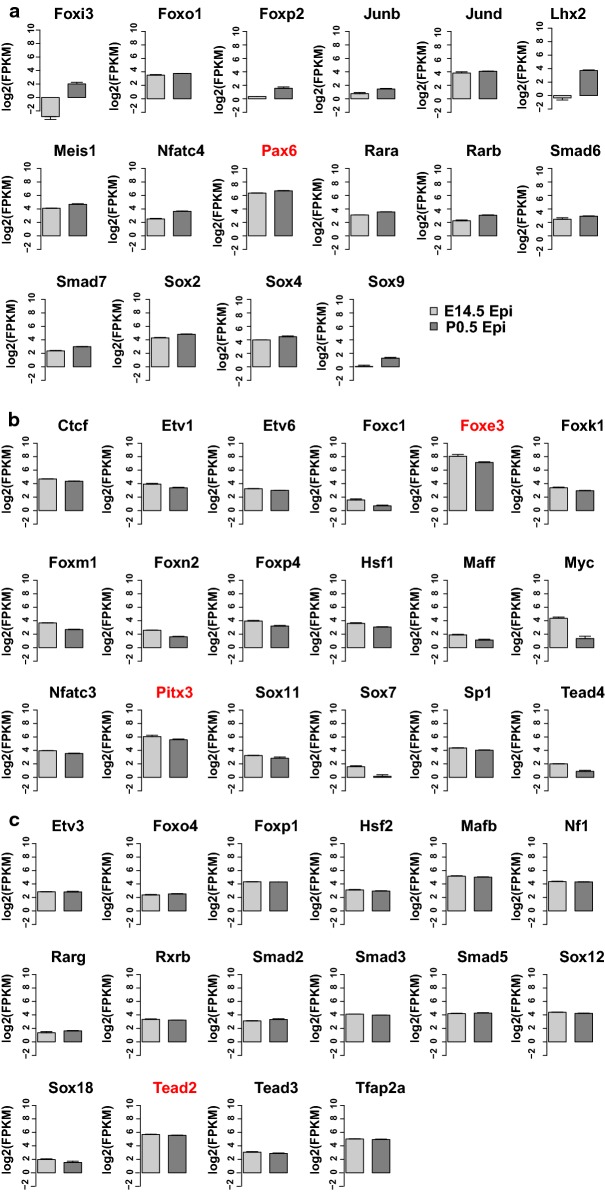
Expression profiles of selected transcription factors linked to lens epithelium maturation (Path2). **a**–**c** Bar plots show FPKMs of the transcription factors linked to epithelium maturation path at E14.5 and P0.5 epithelium. **a** 16 TFs with significantly increased expression from E14.5 epithelium to P0.5 epithelium. **b** 18 TFs with significantly decreased expression from E14.5 to P0.5 epithelium. **c** 16 TFs with stable expression from E14.5 to P0.5 epithelium

Genetic loss-of-function studies in the mouse lens have been published for the most abundant lens transcription factors, evaluated by their steady-state mRNA levels (FPKM values > 50), including *c*-*Maf* [34–36], *Prox1* [32, 37], *Hsf4* [38, 39], *Sox1* [40], compound *MafG*/*MafK* [41], and *N*-*Myc* [42]. In contrast, transcription factors identified in Path2 were divided into three groups (16, 18, and 16 TFs), including genes with up- and down-regulated expression (Fig. 6; Foxj2 mRNAs are not shown due to low expression). These groups are comprised of TFs with both established and potentially novel roles in lens development. Among them, loss of function of Pax6 [43], Foxe3 [44, 45], and Pitx3 [46] are known to cause major lens defects. The function of abundant TF Tead2 in lens development needs further exploration. These predicted *cis*-sites are recognized by a number of well-established transcription factors that regulate lens differentiation [11] and specific examples of promoters and enhancers (i.e., Foxe3, Prox1, and Mip) are summarized in Discussion. Taken together, these results provide direct links between enriched *cis*-motifs in open chromatin domains and up-regulation of specific transcription factors, including those with proven roles in lens differentiation.

At the individual gene and locus level, we highlighted specific examples of open chromatin regions at potential enhancers of genes encoding key lens transcription factors and structural genes (Additional file 10: Fig. S7 a and b). *Foxe3* and *Myc* loci had open chromatin regions upstream of their transcription start sites. *Etv1, Pitx3, Rxra, Hsf4, Nfat5, Etv5, Tead1, Meis2, Prox1*, and *Sox1* loci also display open chromatin regions inside of their gene bodies. In all instances, the open chromatin regions overlap with evolutionarily conserved noncoding sequences, making them strong candidates for distal enhancers. Likewise, we found potential distal enhancers for *Lim2*, *Mip*, and *Sptbn1*, all of which encode important lens structural proteins. Collectively, these results underscore the values of present ATAC-seq data in providing novel comprehensive information for predicting both lens promoters and enhancers.

### Pax6 bound at both open and closed chromatin regions in lens and forebrain

**Fig. 7:**
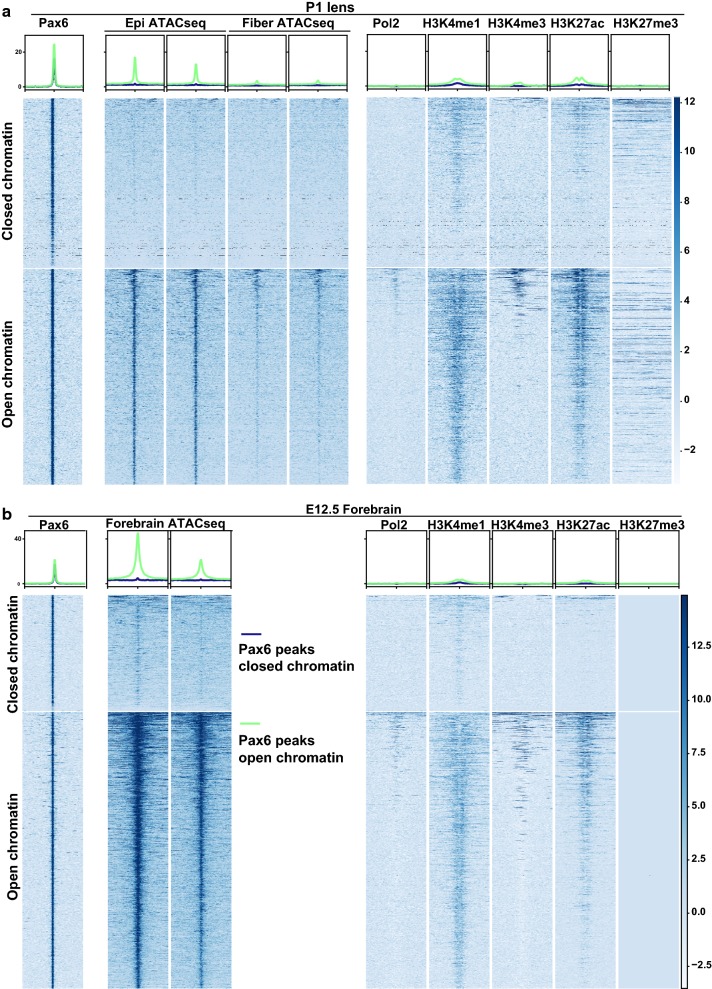
Pax6 binding to both open and closed chromatin regions. **a**, **b** Heat maps show the signals of ATAC-seq (only P0.5 lens epithelium and fibers ATAC-seq data were shown for lens) and ChIP-seq within ± 5 kb from Pax6 peak centers of lens (**a**) and E12.5 forebrain (**b**). The blue and green lines at the top represent mean read densities of the corresponding ATAC-seq or ChIP-seq at Pax6 peaks at closed and open chromatin, respectively

Ideally, it would be important to compare our ATAC-seq data with ChIP-seq data of lens TFs, but so far only Pax6 has been analyzed by ChIP-seq using newborn whole lenses [16]. Interestingly, we found 2124 (56%) Pax6 peaks at open chromatin regions (i.e., overlapping with ATAC-seq peaks) and a comparable number of 1688 (44%) at closed chromatin regions (i.e., not overlapping with ATAC-seq) in lens samples. Analysis of the ATAC-seq and ChIP-seq signals demonstrated clearly that Pax6 can bind in vivo to both open and closed chromatin regions (Fig. 7a). In support of this, we further examined Pax6 binding in mouse forebrain and found 1044 (30%) of the 3536 Pax6 peaks at closed chromatin regions (Fig. 7b). For example, Pax6 peaks at the *Prox1* locus were located at open chromatin regions, but the Pax6 peak at the *Hivep2* locus in lens was located at closed chromatin regions (Additional file 11: Fig. S8a). Additionally, we examined 436,176 peaks from the mouse chromatin accessibility atlas generated with single-cell ATAC-seq from 13 different mouse tissues [47]. Even at this scale, we found that 54% (2064 Pax6 peaks) and 47% (1669 Pax6 peaks) of Pax6 peaks in both lens and forebrain were at closed chromatin regions (Additional file 11: Fig. S8b) using a range of tissues. Collectively, these findings support a model in which Pax6 can bind to both open and closed chromatin regions.

### Pax6 binding at open and closed chromatin regions is related to distinct functions

**Fig. 8:**
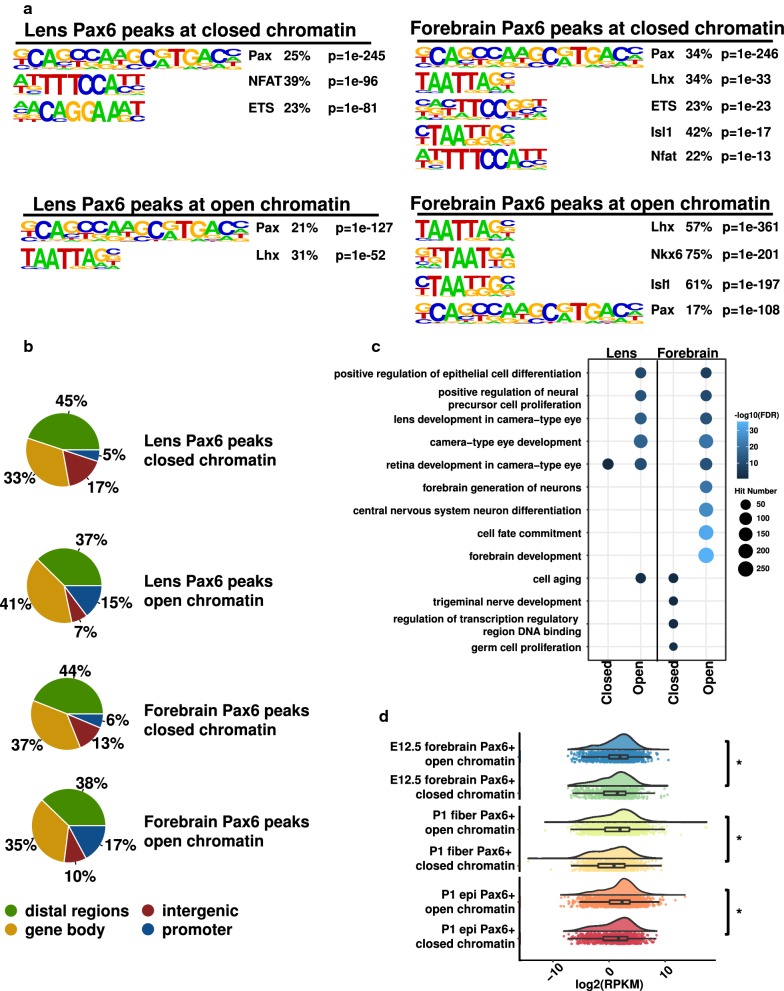
Motif and function analysis of Pax6 binding sites at open and closed chromatins. **a** Top enriched motifs at Pax6 binding sites at open and closed chromatins in lens and E12.5 forebrain. **b** Pie charts show the genomic distributions of the Pax6 peaks at open and closed chromatin in lens and E12.5 forebrain. **c** Dot plot shows enriched GO terms for Pax6 peaks at open and closed chromatins in lens and E12.5 forebrain. The dot color represents –log10(FDR), and the dot size represents number of regions in the GO terms. **d** Raincloud plots show the expression of genes with Pax6 peaks at open and closed chromatin regions in P1 epithelium and fibers as well as E12.5 forebrain. The * indicates *p* < 0.05 by Mann–Whitney U test

An active area of TF–chromatin interaction research is which transcription factors possess the “pioneering” activity to open closed chromatin [48] and the molecular mechanisms governing dual functions of a large number of transcription factors in gene activation and repression [49–51]. To address these issues, we employed ChIP-seq datasets, including Pax6, RNA polymerase II (Pol2), and histone modifications [16]. Despite little difference in Pax6 signals between Pax6 binding sites at open and closed chromatins, we found that Pax6 binding at open chromatin regions had higher signals of Pol2, H3K4me1, H3K4me3, and H3K27ac than at closed chromatin in both lens and forebrain (Fig. 7). In support of *bona fide* binding at the closed chromatin, we found that the Pax6 binding motif was similarly enriched at the Pax6 peaks in both open and closed chromatin regions (Fig. 8a), using HOMER (v 4.7) [31]. The motif analysis, however, found that the ETS motif was only enriched at Pax6 peaks at closed chromatin in lens and forebrain (Fig. 8a). An examination of the distributions of Pax6 peaks showed that the Pax6 peaks at open chromatin regions in both lens and forebrain had much higher percentages of promoter peaks (*p* = 0.03 for percentages of promoter peaks in open vs. closed chromatin in lens, and *p* = 0.03 for open vs. closed chromatin in forebrain; Chi-squared test) (Fig. 8b). Moreover, we found that the Pax6 peaks at open and closed chromatin regions were enriched for different GO terms (Fig. 8c). For example, Pax6 binding at open chromatin regions in lens and forebrain was enriched for “lens development in camera-type eye,” “forebrain development,” and “forebrain generation of neurons,” respectively. However, Pax6 binding at closed chromatin regions in lens and forebrain was not enriched for any tissue development associated GO terms.

We further evaluated the expression levels of Pax6 bound genes in lens and forebrain at open and closed chromatin. We found that genes with Pax6 binding at open chromatin regions exhibited significantly higher expression than genes with Pax6 binding at closed chromatin regions in P0.5 lens epithelium, P0.5 lens fibers, and E12.5 forebrain [16] (Mann–Whitney *U* test, *p *< 0.05; Fig. 8d). Using differentially expressed genes upon conditional depletion of *Pax6* in the lens [52], we then probed how the two different Pax6 binding modes were related to its active or inhibitory functions. We found that genes with Pax6 binding at closed chromatin regions were significantly more likely to be inhibitory targets of Pax6, i.e., up-regulated upon *Pax6* depletion compared to Pax6 sites located at open regions (*p* < 0.05, Chi-squared test, Additional file 11: Fig. S8c).

Enrichment of histone acetyltransferase P300 is a hallmark of active chromatin, including “super-enhancers” [53]. Both P300 and CBP are required for lens placode induction [54]. Pax6 can form a complex with P300 in both pancreas [55] and lens [17]. To explore the relationship between Pax6 and P300 binding, we analyzed previously published P300 ChIP-seq data in E11.5 forebrain [56]. We found that a total number of 724 P300 peaks overlapped with Pax6 binding sites at open chromatin regions, but only three P300 peaks overlapped with Pax6 binding sites at closed chromatin (Additional file 11: Fig. S8d). Collectively, these findings strongly suggest two distinct Pax6 binding and functional mechanisms at open and closed chromatin regions.

### Pax6 binding at open chromatin regions in lens epithelium and fibers

**Fig. 9:**
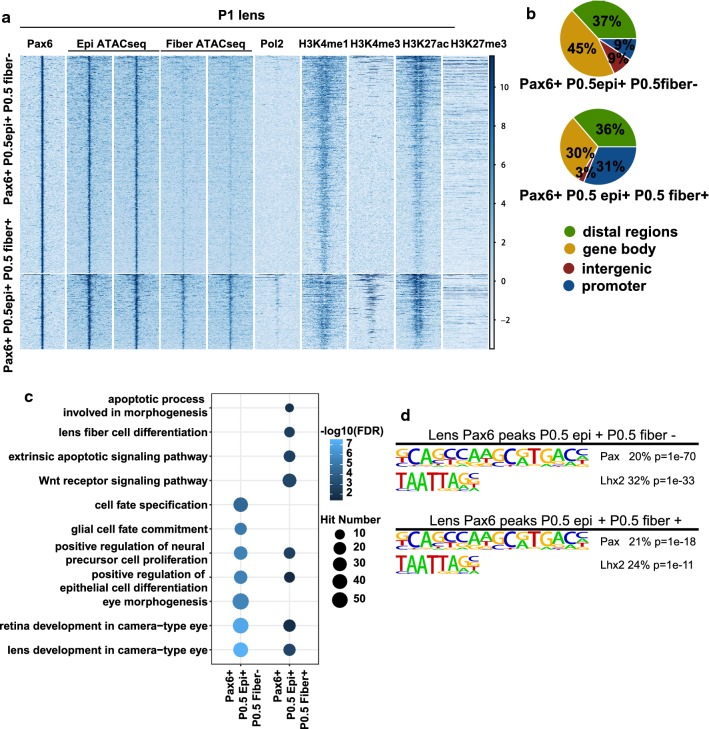
Motif and functional analysis of Pax6 binding sites with two chromatin patterns at P0.5 lens. **a** Heat map shows signals of ChIP-seq and ATAC-seq within 5 kb from Pax6 peak centers at open chromatin regions in P0.5 lens epithelium (Pax6 + P0.5 epi + P0.5 fiber-) and at both P0.5 lens epithelial and fiber open chromatin regions (Pax6 + P0.5 epi + P0.5 fiber +). **b** Pie charts show the genomic distributions of the two groups of Pax6 peaks. **c** Dot plots show the enriched GO terms for the two groups of Pax6 peaks. The dot color represents –log10(FDR), and the dot size represents number of regions in the GO terms. **d** Top enriched motifs inside the groups

As Pax6 was previously shown to function differently in epithelium and fibers during lens development [57], we further examined chromatin accessibilities of the Pax6 binding sites, identified by ChIP-seq, at P0.5 lens epithelium and fibers. In summary, we identified 1771 Pax6 peaks that were located to open chromatin regions only in P0.5 epithelium, 463 to open chromatin regions in both P0.5 lens epithelium and fibers, and 30 to open chromatin regions only in P0.5 fibers. The first two groups are shown Fig. 9a. We found that the peaks located to open chromatin regions in both lens epithelium and fibers had higher RNA polymerase II and H3K4me3 signals and a higher percentage of peaks at promoter regions (*p* = 0.0002, Chi-squared test), compared to the group with open chromatin regions only in lens epithelium (Fig. 9b). HOMER motif analysis identified that only Pax and Lhx motifs were enriched in these two groups of Pax6 peaks (Fig. 9d). Based on these results, we further analyzed the GO terms enriched in the two groups of Pax6 peaks using the GREAT software [30]. Except for the shared GO terms on lens development and epithelial cell differentiation, we noted that Pax6 peaks at open chromatin regions shared in P0.5 epithelium and P0.5 fibers were enriched for “Wnt receptor signaling pathway,” “apoptotic process involved in morphogenesis,” and “lens fiber cell differentiation” (Fig. 9c). We found 20 “Wnt receptor signaling pathway” related genes with Pax6 binding at open chromatin regions, such as *Wnt7b*, *Tle1*, *Tle4*, and *Gsk3a*. These results further support the model in which Pax6 actively regulates Wnt signaling in both lens epithelium and fibers, as indicated in earlier studies [57–59].

## Discussion

A major question in the area of developmental biology, gene expression, genomics, and epigenomics is to understand how *cis*-genomic information is organized as chromatin and how local and global chromatin structure regulates individual gene expression. In the present study, we examined changes in chromatin structure and mRNA levels during lens differentiation, focusing on two differentiation pathways: lens epithelium → advanced lens fibers → maturing lens fibers (Path1) and early lens epithelium → maturing lens epithelium (Path2). Based on the genome-wide correlations of open chromatin and steady-state mRNA levels, the present study unravels three principles of lens-specific gene regulation that are generally applicable to other tissues. First, *cis*-regulatory logic of promoters and enhancers emerges from commonly enriched *cis*-motifs as well as expression dynamics and abundance of individual DNA-binding transcription factors. Second, identification of active promoter and enhancer regions in lens, coupled with evolutionarily conserved noncoding sequences, enables the identification of noncoding mutations and their roles in lens diseases, including cataract and presbyopia. Third, distinct patterns of Pax6 binding in open/H3K27ac^+^/P300^+^ marked and closed chromatin regions provide novel insights into molecular mechanisms of this key regulatory factor of embryonic lens development.

Despite the progress, there are several limitations in the study. First, we found that many differentially accessible regions were not assigned to differentially expressed genes. This could be caused by a plethora of transcriptional regulatory mechanisms. For example, multiple enhancers can exist for genes and changes of chromatin accessibility for a single enhancer may not be sufficient to elicit any significant mRNA level changes. Additionally, some enhancers might locate at distal regions even beyond multiple proximal genes and therefore could not be assigned to the correct target genes based on the current annotation strategy. It is also possible that TFs residing in DARs require activation through extracellular signaling and/or presence of additional activators. Another limitation is the *cis*-motif analysis. The whole analysis is highly dependent on the current knowledge of TFs and will be affected by the lack of knowledge for some TFs, such as Prox1 (see below for details) and Pknox1 [60]. Additionally, motifs for some important lens TFs, such as Oct1 (Pou2f1) [61] and Six3 [62], were detected in our motif analysis but their low ranking masked their potential importance, as we focused on high-ranked TFs in the current study. Lastly, although it is exciting to find that Pax6 can bind to open and closed regions, this observation needs to be further tested experimentally since our current ATAC-seq might not have identified all the accessible chromatin regions in lens. Nevertheless, the presence of Pax6 binding in closed chromatin in forebrain strongly supports our findings in the lens.

The lens fiber cell differentiation cascade (Path1) is regulated by a well-established group of transcription factors, including c-Maf, Hsf4, Pax6, Prox1, and Sox1, together with signal-regulated transcription factors, nuclear effectors of BMP, FGF, Notch, and Wnt signaling [11]. Analysis of the expression patterns of transcription factors within Path1 shows sustained up-regulation of 11 genes encoding transcription factors (e.g., Hsf4, Maf, Sox1, and Prox1). Two other groups are comprised of three factors that are higher in early compared to later lens fibers (e.g., Foxk2, MafG, and N-Myc) and a group of four TFs marked by “delayed” up-regulation (e.g., Foxo3, MafK, Smad1, and Smad4). Within the first group, genetic loss-of-function data exist for lens morphogenesis defects caused by Sox1 [40], c-Jun [63], Hsf4 [38, 39], and Maf [34–36] genes. Remarkably, their steady-state mRNA levels are the highest among this group examined. Likewise, depletion in lens of Gata3 [64], compound MafG/MafK [41], N-Myc [42], and Smad4 [65] also perturbed lens differentiation. Given the enrichment of motifs and high expression levels of Gatad1 and Tead2, we propose that these transcription factors are excellent candidates to examine their roles in lens differentiation, an idea further supported by their enrichment in lens comparing to the lens-depleted whole embryo from the iSyTE2 database [66]. Tead factors, nuclear effectors of Hippo-Yap signaling, bind to non-phosphorylated Yap, and FGF signaling increases cytoplasmic levels of phosphorylated Yap [67]. It is noteworthy that binding sites of a well-known homeodomain-containing factor and regulator of β- and γ-crystallin gene expression, Prox1, were not identified by the current methods, most likely due to its partially established DNA-binding mechanisms [33, 68]. Nevertheless, we indeed detected ATAC-seq peaks at previously identified binding regions of Prox1 in four genes, including *Lctl*, *Fgfr3*, *Fgfrl1*, and *Crybb1* [32, 33] (Additional file 9: Fig S6).

The lens epithelium differentiation–maturation pathway (Path2) remains poorly understood compared to Path1. Unlike the terminally differentiated fiber cells, the lens epithelium contains the stem cell-like Sox2^+^ cells which are thought to drive lens growth [69]. Notably, the cell proliferation rates vary both spatially and temporally during lens epithelium maturation [9, 12]. Apart from Pax6, three prominent transcription factors involved in lens epithelium morphogenesis are AP-2α [70], FoxE3 [44, 71, 72], and c-Myc [73, 74]. The RNA-seq data show up-regulation of Pax6, Sox2, and Meis1 during lens epithelium maturation. These factors form the earliest network of genes to generate lens progenitor cells [75–78]. In contrast, down-regulation of three lens TFs, AP-2α [70, 79], c-Myc [74], and Pitx3 [46, 80–82] is consistent with reduced proliferation during lens epithelium maturation. The present data unravel dynamics of nearly 20 novel DNA-binding transcription factors that are either up- or down-regulated during lens epithelium terminal differentiation. Among this group, expression data prioritize studies of Tead2, Tead3, and Tead4 as these three factors function within Hippo-Yap signaling, which has been shown to control lens epithelial cell proliferation, polarity, and tension through actomyosin networks [83, 84].

**Fig. 10:**
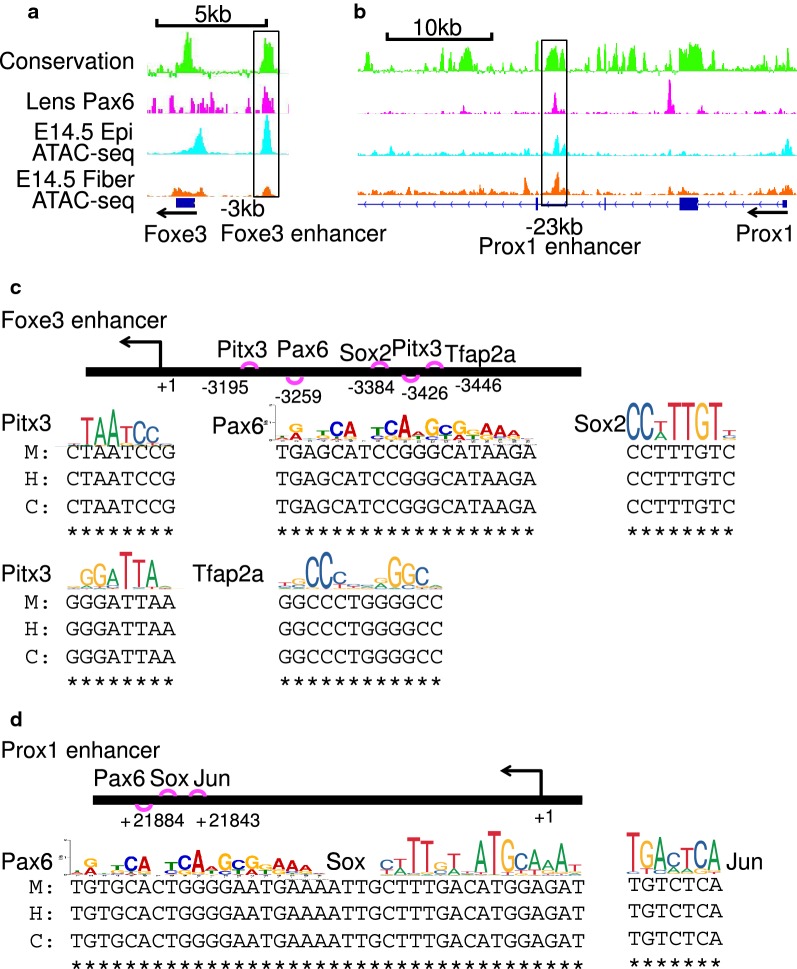
Examples of clusters of predicted *cis*-regulatory sites in Foxe3 and Prox1 enhancers. **a** *Foxe3* locus including its 5′-enhancer (− 3 kb). **b** *Prox1* locus including its 3′-distal enhancer (− 23 kb). **c** Experimentally validated (Pitx3) and predicted (Sox2 and Tfap2a) grammar of the Foxe3 enhancer. **d** Experimentally validated (Pax6) and predicted grammar (Sox and AP1/Jun) of the Prox1 enhancer. M, H, and C show DNA sequences from mouse, human, and cow, respectively

**Fig. 11:**
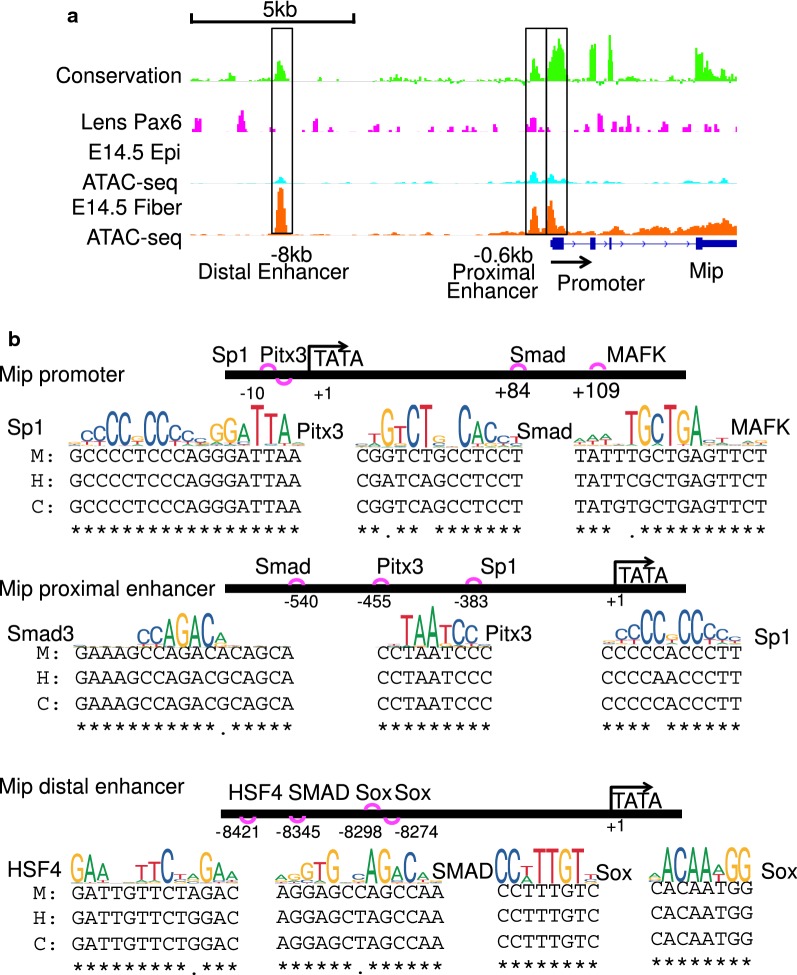
Examples of clusters of predicted *cis*-regulatory sites in Mip promoter and its proximal and distal enhancers. **a** *Mip* locus and its promoter and two enhancers (− 0.6 kb proximal and − 8 kb distal). **b** Experimentally validated (Pitx3 and Sp1) and predicted (Hsf4, MafK, Smad, and Sox) grammar of the Mip promoter and enhancers. M, H, and C show DNA sequences from mouse, human, and cow, respectively

To illustrate how current unbiased predictions correlate with experimental data, we use *Foxe3*, *Prox1*, and *Mip* loci as examples (Figs. 10, 11). We show evolutionarily conserved sites and comment on the experimental validation by previous studies. Foxe3 is an abundant DNA-binding transcription factor in lens epithelium [44, 71, 72]. A Foxe3 enhancer [85] is predicted here to possess two Pitx3-binding sites (Fig. 10), which is in agreement with earlier experimental studies [82]. Our data predict a distal Prox1 enhancer including an evolutionarily conserved Pax6-binding site (Fig. 10); this is supported by previous Pax6 ChIP-seq data [16]. Our studies identify the promoter [86–88] as well as a pair of proximal and distal enhancers for *Mip* locus (Fig. 11). Our data predict both Pitx3- and Sp1-binding sites as shown experimentally earlier [86, 88]. Future studies will be aimed to dissect temporal and spatial activities of these individual regulatory regions in *Foxe3*, *Prox1*, and *Mip* loci and identify specific roles of predicted individual *cis*-sites, as we described earlier using the c-Maf and Cryaa promoters and enhancers [89, 90].

The earliest studies of promoters and enhancers employed systematic dissections of 5’-promoter flanking regions coupled with evaluation of distal enhancer effects using reporter assays and transiently transfected cells. With the dawn of complete genomic DNAs of model organisms, the use of evolutionarily conserved noncoding regions paved the road to identify a large number of enhancers in numerous tissues, including the lens [78, 89, 91]. More recently, enhancers were also predicted from a combination of specific histone modifications, such as H3K4me1 and H3K27ac [92]. Nevertheless, the main challenge of studies involving primary tissues was both the quantity and quality of the starting materials for histone PTMs, requiring millions of cells per experiment. Introduction of ATAC-seq greatly simplified such studies as it can be performed with much smaller numbers of cells [18]. A number of genes have been identified that cause human cataracts as well as lens opacities in model organisms [66, 93, 94]. Nevertheless, the only noncoding mutations are known in 3′-distal enhancer of the PAX6 locus [95, 96]. The present studies thus provide critical starting points [97] for screening of mutations in the enhancers and promoters of genes encoding known cataract-linked genes [93] and in genes that predict cataract-causing genes using their enrichments over non-lens tissues as listed in the iSyTE databases [66, 94].

Pax6 is essential for multiple stages of lens formation, starting with lens induction [54, 75, 98–100], through proliferation of the lens placode [101], lens placode invagination and separation of the lens vesicle from the surface ectoderm [43, 102, 103], cell cycle exit to form primary lens fiber cells [57], crystallin gene expression [89], and degradation of nuclei in maturing lens fibers [104, 105]. These functions can be explained from the perspective of evolution, the “intercalary” hypothesis [106], and shared motifs between Pax6-binding sites with other commonly used cis-motifs recognized by heat shock factors, antioxidant response element, and half of p53-binding site [107]. The present data demonstrate that Pax6 can bind to both open and closed chromatin domains. This is consistent with previous studies of Pax6 complexes with different activities to augment and/or repress transcription [17]. We propose that posttranslational modifications of Pax6, particularly in its C-terminal domain [108, 109] may select appropriate chromatin remodeling complexes, but more studies are needed to address this. This notion is further supported by recent studies of structurally similar Pax7 in vivo binding patterns detected in both open and closed chromatin domains [110].

## Conclusions

Our study provides the first in-depth analysis of open and closed chromatin dynamics coupled with mRNA changes during mammalian lens differentiation. The results reveal groups of co-regulated genes required for lens fiber cells and lens epithelium differentiation and maturation. The molecular base of the co-regulation is illuminated by an unbiased discovery of highly enriched motifs in putative promoters and enhancers, which are comprised of arrays of *cis*-sites known to bind both established transcription factors in lens differentiation (e.g., c-Maf, Pax6, and Sox1) and novel candidate factors (e.g., Tead2, Tead4, CTCF, Gatad1, and NF1). The present studies also uncover novel molecular functions of Pax6, namely its presence in both open and closed chromatin domains, marked by differential levels of histone modifications, consistent with its dual roles as transcriptional activator and repressor. Use of other vertebrate model organisms, such as chicken, provides comparative insights into the chromatin changes during lens cellular differentiation [111]. Collectively, these studies pave the road for follow-up functional studies to dissect roles of candidate enhancers in temporal and spatial gene regulation during lens development and identification of mutations in noncoding regulatory regions of genes that regulate lens development and when mutated could cause cataracts and other lens abnormalities in humans.

## Materials and methods

### Tissue samples and ATAC-seq

Mouse lenses from E14.5 to P0.5 CD1 mice (Charles River) were micro-dissected under the microscope into epithelium and fibers as described earlier [112, 113]. Six P0.5 and eight E14.5 lenses were used per sample. After dissociation, cells were resuspended in cold PBS. Following the ATAC-seq protocol [18], approximately 50,000 P0.5 epithelial, 25,000 P0.5 fiber, 15,000 E14.5 epithelial, and E14.5 fiber cells were used for cell lysis and transposase (2.5 μl transposase in 50 μl buffer) treatment at 37 °C for 30 min. The cell numbers for the experiments were carefully optimized through several trials to achieve clear nucleosome patterns and ensure the qualities of the libraries. The DNA fragments were then purified using MinElute PCR Purification Kit (Qiagen, Cat No. 28004) and amplified by PCR. Both quality and quantity of ATAC-seq libraries were examined by Bioanalyzer and qPCR. The ATAC-seq libraries were sent for 75-bp paired-end sequencing on Illumina NextSeq 500.

### Lens ATAC-seq data analysis and external ATAC-seq data

The 75-bp paired-end ATAC-seq reads were first trimmed by trim-galore (version 0.4.1, https://www.bioinformaticsbabrahamacuk/projects/trim_galore/) to remove adaptors and then mapped to the mouse genome (mm10) using Bowtie2 (version 2.3.3.1) [114] with “-X 2000 –no-mixed –no-discordant –local” parameters. After filtering mitochondrial and duplicated reads through SAMtools (v 1.2) [115] and picard (v 2.1.1, https://www.broadinstitutegithubio/picard/), we used MACS2 (v 2.1.0) [23] for peak calling, with parameters “-f BAMPE -g mm -q 0.05.” After merging the peaks called from individual samples with bedtools2 (v 2.26.0) [116] (parameter ‘-d 10’) and filtering mm10 blacklist regions [24], we identified 185,297 non-redundant ATAC-seq peaks in total. The dataset supporting the conclusions of this article is available in the GEO repository, number GSE124497 (https://www.ncbi.nlm.nih.gov/geo/query/acc.cgi?acc=GSE124497). The fastq files for E12.5 and E14.5 forebrain (ENCSR559FAJ, ENCSR810HQR, generated by Bing Ren laboratory from UCSD), E14.5 liver (ENCSR032HKE, generated by Bing Ren laboratory from UCSD), and ESCs ATAC-seq (GSE66390) [26] data were downloaded from the ENCODE [24, 27] and GEO [28] and processed through the same pipeline for the analysis. The mouse ATAC-seq peak file (mm9) across 13 tissues from 8 weeks mice was downloaded from the GEO (GSE111586) [47] and converted to mm10 coordinates using the LiftOver tool in the UCSC genome browser [117].

### Peak annotation and data visualization

The peaks were associated with genes in the Refseq annotation downloaded from the UCSC genome browser [117] in June 2018. The peaks were assigned to genes in a stepwise manner: to promoter regions (< ±2 kb of transcription start sites), gene body, distal regions (< ±50 kb from the gene body), and otherwise intergenic regions. The IGV (Integrative Genomics Viewer, v 2.4.7) software [118] was used to visualize ATAC-seq and ChIP-seq signals at individual regions. After normalizing the samples to the same sequencing depth, deepTools2 (v 2.5.2) software [119] was used to plot the heat maps to show signals around peak regions with default parameters.

### Identification of unique and shared differentially accessible regions

The read pair numbers inside each ATAC-seq peak were calculated using the HTseq (v 0.8.0) [120] with parameter “–nonunique all.” The differentially accessible regions were identified using EdgeR (v 3.22.3) [25] with the cutoff of cpm > 2, FC > 2 and FDR < 0.05 through pairwise spatial and temporal comparisons (E14.5 epithelium vs. E14.5 fibers, P0.5 epithelium vs. P0.5 fibers, E14.5 epithelium vs. P0.5 epithelium, E14.5 fibers vs. P0.5 fibers). One peak could be present in multiple DAR lists if its signal changes met the above criteria in those comparisons. Four unique clusters and four shared clusters were selected from the unique and shared peaks using bedtools (v2.23.0). For example, E14.5 epithelium unique peaks were selected from the DARs with higher signals in E14.5 epithelium by intersection E14.5 epithelium versus E14.5 fibers and E14.5 epithelium versus P0.5 epithelium.

### Identification of DARs at DEGs

The differentially expressed genes were obtained from the E14.5 and P0.5 lens RNA-seq data at FDR < 0.05 [29]. Here, we only analyzed DARs at DEGs when the ATAC-seq signal changes in the DARs were in the same directions as the expression changes of the DEGs. Such DARs at DEGs were then combined into two paths (lens fiber cell differentiation path and lens epithelium maturation path) for lens cells differentiation. The lens fiber cell differentiation path included all the DARs from E14.5 epithelium versus E14.5 fibers and E14.5 fibers versus P0.5 fibers. The lens epithelium maturation path included all the DARs from comparison of E14.5 epithelium versus P0.5 epithelium. We further divided them into clusters by hierarchy clustering methods. The mean FPKMs were used for quantification of gene expression.

### Motif analysis and function analysis

The enriched motifs inside each cluster of peaks were identified using the HOMER software (v 4.7) [31]. For ATAC-seq peak clusters, all ATAC-seq peaks were used as the background for motif scan. For ChIP-seq peak groups, random genomic background was used. We further selected the top non-redundant enriched motifs for discussion. For example, PAX5 and Pax8 motifs were listed as PAX motif in Figs. 3 and 4 as binding sites of Pax6 are similar to other binding sites of Pax3/7 and Pax5 [16]. The GREAT software (v 3.0.0) [30] was used to find the enriched GO terms for each cluster of peaks. We selected the promoters and enhancers in lens important genes, such as *Mip*, *Foxe3*, and *Prox1*, and used the FIMO software [121] to scan them against the motifs enriched at DARs of DEGs. The sequence conservation of the motif sites was then checked by the conservation data downloaded from UCSC genome browser [117] (mm10.phyloP.60way). We further extracted the corresponding human and cow sequences by VISTA software [122] and aligned them using MAFFT with default settings [123].

### ChIP-seq data and analysis

Pax6, Pol2, H3K4me1, H3K4me3, H3K27ac, H3K27me3 and inputs ChIP-seq fastq and bed files in P1 lenses and E12.5 forebrain were downloaded from the GEO (GSE66961) [16, 28]. The reads were aligned to the mouse genome (mm10) using Bowtie2 (version 2.3.3.1) [114] with default parameters. Duplicated reads were removed using SAMtools (v 1.2) [115], and deepTools2 (v 2.5.2) [119] was further used to subtract input signal and normalize the reads to the same sequencing depth. P300 ChIP-seq peaks at E11.5 forebrain were downloaded from the supplementary files in the paper [56]. All peak files were converted from mm9 to mm10 using LiftOver tool from UCSC genome browser [117] when necessary.

## Additional files

**Additional file 1: Table S1.** Summary for the ATAC-seq data.

**Additional file 2: Fig. S1.** Quality controls of ATAC-seq data. **a.** Distributions of insert sizes from 8 ATAC-seq libraries. **b.** Heatmap shows Pearson correlation coefficients among samples, computed from reads mapped to peaks. **c.** Scatterplots of mean normalized counts (mean read counts inside peaks from biological replicates normalized by the total read numbers for all peaks) between pairwise spatial and temporal comparisons.

**Additional file 3: Table S2.** Numbers of DARs and DARs at DEGs from each comparison group.

**Additional file 4: Fig. S2.** Overlaps between DARs from 8 comparison groups.

**Additional file 5: Table S3.** Number of peaks inside unique and shared clusters.

**Additional file 6: Fig. S3.** Analysis of unique or shared clusters of open chromatin regions and their corresponding gene expression levels. **a. b.** Heatmaps show ATAC-seq signals and corresponding gene expression for unique and shared peak groups.

**Additional file 7: Fig. S4.** Correlation of DARs and DEGs. **a.** Pie charts show the percentages of differentially expressed genes (DEGs, dark blue), reversely differentially expressed genes (DEGs reverse, light blue) and not differentially expressed genes (not DEGs, red) associated with each group of differentially accessible regions (DARs). **b.** Bar plots show the percentages of DEGs and DEGs reverse in each group of DARs mapped genes divided by the number of peaks annotated to the genes.

**Additional file 8: Fig. S5.** Hierarchical clustering of transcription factors predicted for lens fiber cell differentiation and lens epithelium maturation paths. **a.** 27 TFs showed higher expression in Path1. **b.** 51 TFs showed higher expression in Path2.

**Additional file 9: Fig. S6.** ATAC-seq peaks at previously described Prox1 binding regions at Lctl, Fgfr3, Fgfrl1, and Crybb1.

**Additional file 10: Fig. S7.** Potential enhancers at key transcription factors and structural proteins in lens. **a. b**. Examples of putative enhancers for lens transcription factors (**a**) and structural proteins (**b**) based on ATAC-seq enrichment. The evolutionary conservation (cons) tracks are shown in green. ATAC-seq data are from mouse E14.5 lens epithelium, lens fibers, forebrain, liver, and ESCs.

**Additional file 11: Fig. S8.** Pax6 binding sites at open and closed chromatin play distinct roles for gene expression. **a.** Examples of P1 lens Pax6 peaks at open chromatin regions in Prox1 (left) and closed chromatin regions in Hivep2 (right). **b.** Table shows the number of Pax6 peaks detected at open and closed chromatin regions in mouse ATAC-seq atlas. **c.** Venn diagram shows the overlaps of genes with lens Pax6 peaks at open and closed chromatin regions and the differentially expressed genes in Pax6 KO vs WT. **d.** Venn diagram shows the overlap of Pax6 peaks at E12.5 forebrain and P300 peaks (E11.5 forebrain).

## Abbreviations

ATAC-seq: assay for transposase-accessible chromatin with high-throughput sequencing
DARs: differentially accessible regions
DEGs: differentially expressed genes
ESCs: embryonic stem cells
FDR: false discovery rate
FC: fold change
GO: gene ontology
ChIP: chromatin immunoprecipitation
HAT: histone acetyltransferase
PTM: posttranslational modification
TF: transcription factor
